# Neutrophilic erythrophagocytosis in a child with paroxysmal cold hamoglobinuria

**DOI:** 10.1002/jha2.596

**Published:** 2022-10-18

**Authors:** Omer Pervaiz, Eleni Louka, John Willan

**Affiliations:** ^1^ Department of Haematology Oxford University Hospitals NHS Foundation Trust Oxford UK; ^2^ Department of Haematology Wexham Park Hospital part of Frimley Health NHS Foundation Trust Slough UK

1

A 3‐year‐old previously healthy male child presented to the emergency department with a 2‐day history of fatigue, jaundice, and dark‐brown colored urine following a week of vomiting and fever. There was no significant past medical, drug or family history. Clinical examination was normal apart from jaundice and mildly enlarged spleen on physical examination, confirmed on ultrasonography. Blood tests revealed hemoglobin of 57 g/L, haptoglobin of <0.2 g/L bilirubin of 80 μmol/L, lactate dehydrogenase of 1902 U/L, and C‐reactive peptide of 126 mg/L. Direct antiglobulin test was strongly positive for C3d only. Donath–Landsteiner (D‐L) antibody positivity was not demonstrated. Urine examination revealed hemoglobinuria. Viral swabs were negative for COVID‐19, respiratory syncytial virus (RSV), influenza A&B, and serology did not demonstrate recent infection by cytomegalovirus, Epstein–Barr virus, or parvovirus B19. Blood cultures were negative.

These results were consistent with auto‐immune hemolytic anemia of intravascular type. His peripheral blood smear (shown in the panel) revealed features of hemolytic anemia with polychromasia, red cell agglutination, and spherocytosis. In addition to this, neutrophilic erythrophagocytosis (Figure 1, blue arrows) can be seen, which is typical in paroxysmal cold hemoglobinuria (PCH), though it can occasionally be seen in other conditions such as myelodysplasia [1].

**FIGURE 1:**
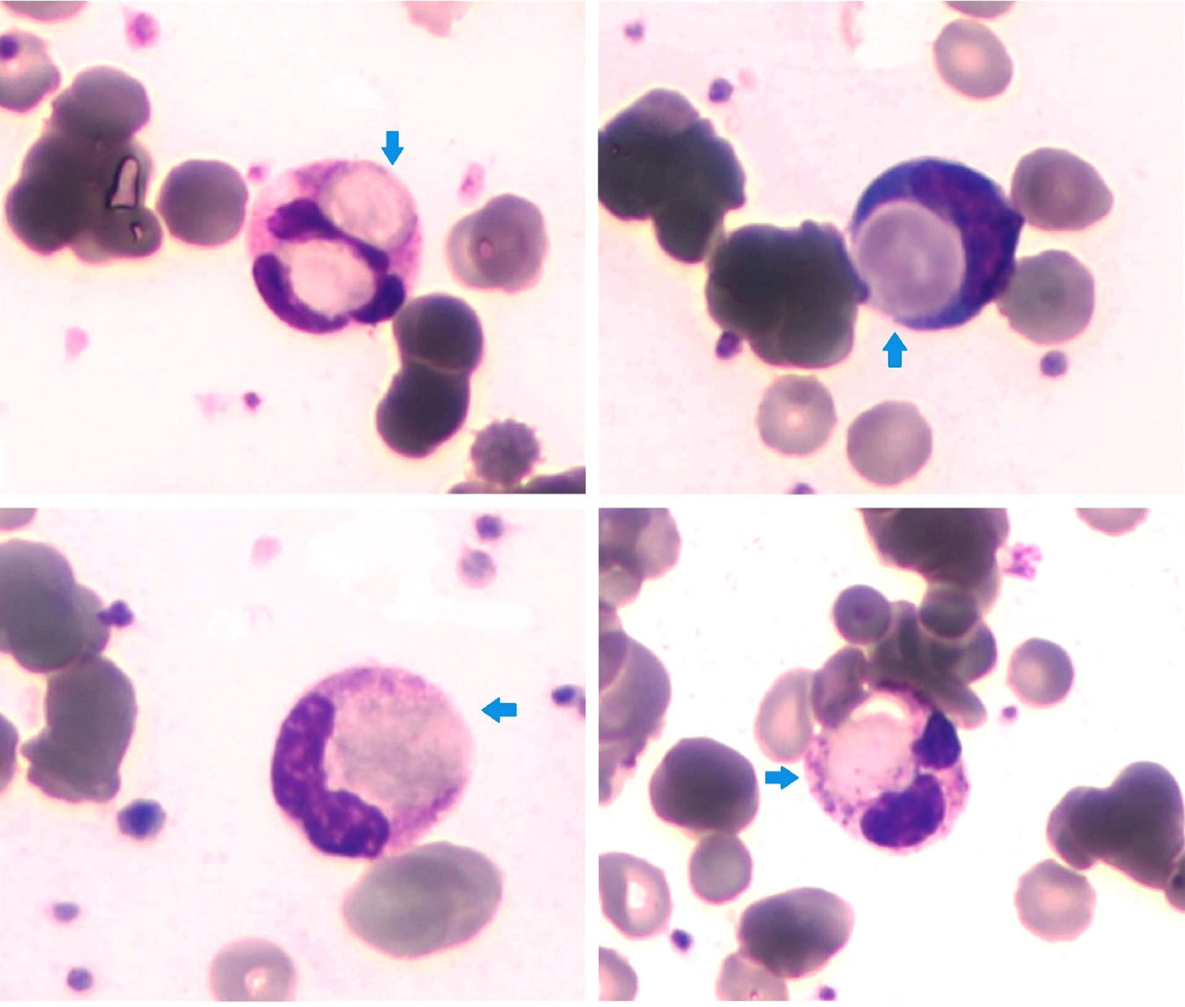
Neutrophils with erythrophagocytosis on peripheral blood smear

A diagnosis of PCH was reached and patient was treated according to the standard protocols with warmed blood transfusions and folic acid. He required multiple transfusions during the first 2 weeks of presentation. He was discharged from the hospital once his hemoglobin stabilized. We continued to monitor his condition as an outpatient for few weeks and his hemoglobin showed a consistent upward trend, completely resolving within 2 weeks of discharge.

Although PCH is a rare disorder, it is one of the most common causes of autoimmune hemolytic anemia in children [2]. Historically, it was often associated with chronic Syphilis infection and patients would experience paroxysms of hemolysis on exposure to cold; however, nowadays, it almost always occurs as acute transient anemia in children after some form of viral illness. As a result, bouts of hemolysis on exposure to cold are now rarely demonstrated [2]. Symptoms are normally transient and require no specific treatment other than supportive measures like transfusion support. Immunosuppression with steroids has not proven to be effective [2].

PCH is classically diagnosed following demonstration of a D‐L antibody. This specialized test is performed in reference laboratories in the United Kingdom. The D‐L antibody is a biphasic, usually IgG antibody, that binds with the “P” antigen on red cells at low temperature and causes complement‐mediated lysis in red cells coated with C3 on rewarming [3]. Difficulty in maintaining the correct temperature during sample collection and handling will lead to a falsely negative result [3], and it is the most likely reason for this result in this case. A negative D‐L antibody test should not dissuade the clinician from a diagnosis of PCH when the clinical features and other tests are otherwise supportive.

## CONFLICT OF INTEREST

The authors declare they have no conflicts of interest.

## FUNDING INFORMATION

The authors received no specific funding for this work.

## CONSENT STATEMENT

Written consent was obtained from the patient's guardian.

## Data Availability

Data are available upon reasonable request from the corresponding author.
